# Optimization of a Ge_2_Sb_2_Te_5_-Based Electrically Tunable Phase-Change Thermal Emitter for Dynamic Thermal Camouflage

**DOI:** 10.3390/ma17071641

**Published:** 2024-04-03

**Authors:** Yufeng Xiong, Guoxu Zhang, Yaolan Tian, Jun-Lei Wang, Yunzheng Wang, Zhuang Zhuo, Xian Zhao

**Affiliations:** 1 Center for Optics Research and Engineering, Key Laboratory of Laser & Infrared System, Ministry of Education, Shandong University, Qingdao 266237, China; 2 School of Information Science and Engineering, Shandong University, Qingdao 266237, China

**Keywords:** dynamic thermal camouflage, thermal emitter, phase-change materials, Ge_2_Sb_2_Te_5_

## Abstract

Controlling infrared thermal radiations can significantly improve the environmental adaptability of targets and has attracted increasing attention in the field of thermal camouflage. Thermal emitters based on Ge_2_Sb_2_Te_5_ (GST) can flexibly change their radiation energy by controlling the reversible phase transition of GST, which possesses fast switching speed and low power consumption. However, the feasibility of the dynamic regulation of GST emitters lacks experimental and simulation verification. In this paper, we propose an electrically tunable thermal emitter consisting of a metal–insulator–metal plasmonic metasurface based on GST. Both optical and thermal simulations are conducted to optimize the structural parameters of the GST emitter. The results indicate that this emitter possesses large emissivity tunability, wide incident angle, polarization insensitivity, phase-transition feasibility, and dynamic thermal camouflage capability. Therefore, this work proposes a reliable optimization method to design viable GST-based thermal emitters. Moreover, it provides theoretical support for the practical application of phase-change materials in dynamic infrared thermal camouflage technology.

## 1. Introduction

Regulation of thermal radiation has significant application value in fields of infrared detection [1,2], thermal camouflage [3,4,5], radiative cooling [6,7,8], thermophotovoltaics [9,10,11], and thermal management [12,13]. Among these, infrared thermal camouflage, which is capable of shielding a target object from detection by matching the target’s thermal radiation to that of its surroundings, has been widely studied [14,15,16,17]. Currently, one of the main challenges faced by infrared thermal camouflage technology is how to realize efficient dynamic regulation of thermal radiation to adapt to various background environments. The radiant power theoretically depends on the emissivity of an object, so dynamic infrared thermal camouflage technology aims to explore efficient solutions to flexibly change the emissivity of the object. Recent investigations have revealed that phase-change materials (PCMs) exhibit promising potential for the active tuning of thermal radiation [5,18,19].

PCMs are substances which can transition their physical state (between a solid and liquid state or between one solid state and another solid state) with a large amount of latent heat being absorbed or released [20,21,22,23]. Here, PCMs refer to chalcogenide semiconductors represented by Ge_2_Sb_2_Te_5_ (GST). At room temperature, these materials demonstrate exceptional stability in both amorphous and crystalline phases, which possess notable distinctions in physical properties such as complex refractive index and resistivity between two phases [24,25,26,27]. Multiple dynamic infrared thermal camouflage devices based on GST have been reported to date. A GST–Au bilayer film was used to control the radiation temperature of the target to match the background temperature by tuning the crystallization fraction of GST [19]. When the annealing time of the GST film was 0 s, 40 s, and 60 s, the sample could blend into a black soot reference with temperatures of 30 °C, 40 °C, and 50 °C in thermal images, respectively. By crystallizing a GST film in a layer-by-layer manner via parallel laser printing, the spatial distribution of emissivity can be tailored, and gradient stepwise emission patterns can be produced [28]. Then, different parts of patterns can achieve thermal camouflage at different background temperatures. Plasmonic thermal emitters based on Au–GST–Au structures could achieve dynamic modulation of the emissivity by exciting different magnetic resonance modes in a GST layer [29,30]. The upper Au layer was designed as a cylindrical metasurface, enhancing the optimization freedom of the thermal emitters. The radiant intensity could be modulated by changing the crystallization fraction of GST, thus achieving thermal camouflage in a wide range of background temperatures [31]. Recently, large-bandgap binary PCMs, such as Sb_2_S_3_ [32,33] and Sb_2_Se_3_ [34,35], have drawn much attention due to their low-loss advantages in visible and near-infrared applications. This makes them promising candidates to preserve the resonance performance of emitters during phase transition.

However, there are still some challenges to be addressed. On one hand, these studies have usually employed thick GST layers so that the issue of heat transfer efficiency and quench rate during the crystallization and amorphization processes were not considered. The amorphization process of GST needs a large quench rate over 10^9^ K/s, which puts forward special requirements for thermal structure design. On the other hand, these reports lack the theoretical prediction and experimental verification of the multiple reversible switching of spectral emissivity. This is mainly due to the improper stimulation method of phase transition. Especially, the amorphization process can only be realized by electric or optical pulses, not by hotplate or annealing ovens. In 2021, Zhang et al. proposed an electrically reconfigurable phase-change metasurface which was wire bonded to a circuit board [36]. By using electric pulses with different voltages and widths to control the phase state of meta-atoms, the metasurface achieves a wide spectral tuning range, large optical contrast, and dynamic optical beam steering. Inspired by these results, we proposed an electrically tunable thermal emitter consisting of a metal–insulator–metal (MIM) plasmonic metamaterial based on GST and theoretically demonstrated its feasibility for dynamic infrared thermal camouflage applications [37]. However, the structural optimization, incident angle tolerance, and polarization property of the proposed thermal emitter need to be studied.

To solve these issues, we provide a detailed optical and thermal simulation procedure to optimize the structural parameters in this paper. The dependence of the emissivity spectrum on film thicknesses, Au pillar diameter, and period is studied by sweeping these parameters in a certain range. The influence of incident angle and polarization angle on the emissivity spectrum of the GST emitter is investigated. The heat generation, conduction, and dissipation processes in the GST film are calculated to analyze the temperature uniformity and quench rate. The difference in radiant intensity between amorphous and crystalline GST emitters is computed at different Au pillar diameters under a temperature range from −50 °C to 100 °C. Then, a chosen structural parameter combination is given, with which the tunable range of radiant intensity is the largest. The dynamic thermal camouflage performance is demonstrated in the vegetation background. This study will provide theoretical support for the feasibility of the reversible phase transition of GST emitters and facilitate the practical application of phase-change materials in infrared thermal emitters.

## 2. Methods

### 2.1. Materials and Structures

**Figure 1:**
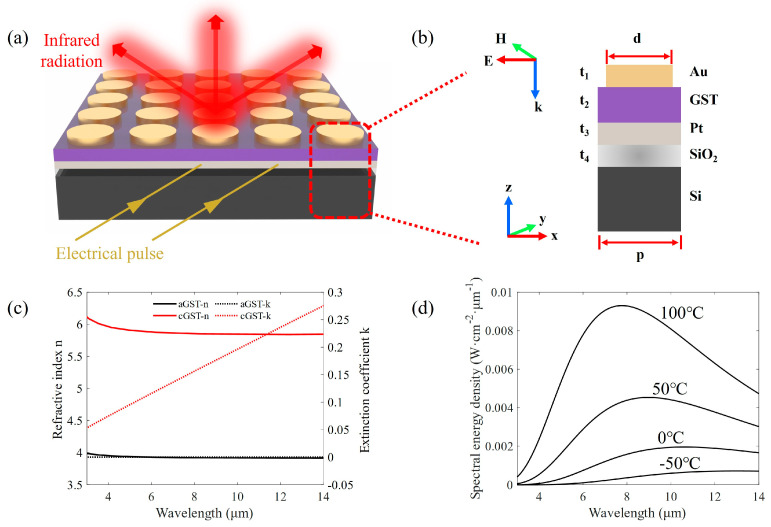
(**a**) Perspective view and (**b**) cross-section view of the electrically controlled infrared thermal emitter based on Ge_2_Sb_2_Te_5_ (GST). The emitter consists of an Au pillar array, GST layer, Pt layer, SiO_2_ layer, and Si substrate. Infrared radiations from the thermal emitter are along the whole hemispherical space, represented by three red arrows. An electrical pulse (yellow arrows) is guided into the Pt layer to generate Joule heat and thus stimulate the phase transition of the GST layer. (**c**) Refractive index and extinction coefficient of GST in 3–14 μm. (**d**) The intensity of spectral radiance from a blackbody with different temperatures.

The core functional structure of the proposed dynamic infrared thermal emitter is an MIM plasmonic metasurface composed of Au–GST–Pt layers. Figure 1a illustrates the structural diagram of the thermal emitter. The MIM layers are deposited on a silicon substrate with an oxide layer (SiO_2_). The oxide layer serves as a thermal insulation layer to improve the heating efficiency. The bottom Pt layer is not only a reflector for infrared radiation but also an electrical microheater to stimulate the phase transition of GST. Electrical pulses are applied to one side of the Pt film, and the other side is grounded. The top Au layer is designed as a cylindrical array to acquire suitable spectral emissivity for dynamic thermal camouflage. Figure 1b shows the cross-sectional view of one unit. The labels t_1_, t_2_, t_3_, and t_4_ denote the thicknesses of the Au pillar, the GST film, the Pt film, and the SiO_2_ film, respectively, and d and p are the diameter and period of the Au pillar, respectively. The electric (E) and magnetic (H) fields are set to be along the *x* and *y* directions, respectively, and the wave vector (k) of the incident light is along the negative *z* direction.

### 2.2. Emissivity and Radiation Intensity

According to Planck’s law, the relationship between the spectral radiance *M_b_* and the wavelength *λ* for a blackbody at a given temperature *T* can be expressed as

(1) $$M_{b}(\lambda ,T)=\frac{2\pi hc^{2}}{\lambda^{5}(e^{\frac{hc}{k\lambda T}}-1)}$$

where *h* is the Planck constant, *c* is the speed of light in a vacuum, and *k* is the Boltzmann constant. Figure 1d shows this relationship over a temperature range from −50 °C to 100 °C. Additionally, the spectral radiance *M_o_* of a real object can be expressed as

(2) $$M_{o}(\lambda ,T)=\varepsilon (\lambda ,T)\times M_{b}(\lambda ,T)$$

where *ε*(*λ*, *T*) means the emissivity of an object. Usually, the effect of temperature on the emissivity can be ignored, i.e., *ε*(*λ*, *T*) becomes *ε*(*λ*). Based on Kirchhoff’s law, the emissivity *ε*(*λ*) is equal to the absorptivity *α*(*λ*) under a thermal equilibrium state. Hence, it can be expressed as

(3) ε(λ)=α(λ)=1−τ(λ)−ρ(λ)

where *τ* (*λ*) is the transmissivity, *ρ* (*λ*) is the reflectivity, and both of them can be obtained from power monitors.

Most commercial thermal imaging cameras determine the radiation temperature of an object by detecting its radiant intensity in an 8–14 μm waveband. The radiant intensity *P*(*T*) is obtained by integrating the spectral radiance in a certain waveband,

(4) $$P(T)=\int_{\lambda_{1}}^{\lambda_{2}}\varepsilon (\lambda )M_{b}(\lambda ,T)d\lambda$$

Here, *λ*_1_ = 8 μm, and *λ*_2_ = 14 μm. It is clear that the radiant intensity *P*(*T*) at a certain temperature is totally determined by the emissivity. Therefore, dynamic thermal camouflage can be achieved by adjusting the emissivity until the radiant intensity of an object is equal to that of the background with different temperatures. In addition, the larger the variation range of the emissivity in 8–14 μm is, the wider the background temperature range that objects can blend into is.

### 2.3. Simulation Modeling

A finite-difference time-domain model was used to simulate the infrared thermal radiation characteristics of the MIM thermal emitter. A plane-wave beam is normal incident to the emitter along the negative *z* directions. Periodic boundary conditions are applied to both the x and y boundaries, and a perfectly matched layer is set to the z boundary. The minimum mesh size is 10 nm in the *z* direction and 15 nm in the *x* and *y* directions. Two power monitors are set up in the plane perpendicular to the *z* axis to record the reflectivity and transmissivity spectra, respectively, which are used to calculate the absorption spectrum. The complex refractive indexes for amorphous GST (aGST) and crystalline GST (cGST) are obtained from Ref. [38] (shown in Figure 1c), and those for other materials are obtained from Palik’s handbook.

To investigate the heat generation, transfer, and dissipation processes of our designed GST emitter under the stimulation of electrical pulses, thermal simulations were performed using a multi-physics finite-element method. An electric current module was used to solve the transient electrical current distribution, and a heat transfer in solids module was employed to simulate the heating transfer and temperature distribution. The two modules were coupled through an electromagnetic heat source model. In the electric current module, an electrical pulse with variable voltage amplitude and pulse duration was connected to the Pt layer. In the heat transfer module, a constant room temperature was adopted for both the side and bottom boundaries, yet thermally insulated boundary conditions were used on the top surface. By taking into account both calculation accuracy and calculation time, the minimum mesh size is about 16 nm in the z direction and about 7.7 um in the x and y directions. The big difference in the minimum mesh size for the three directions is due to the fact that the temperature changes more dramatically in the z direction. To accelerate the calculation, the top Au pillar array was modeled as a homogeneous film, whose accuracy has been verified in simulations. The thermal properties of GST used in the model were derived from Ref. [39]: the thermal conductivities of aGST and cGST are 0.18 W/(m·K) and 1.25 W/(m·K), respectively, and the heat capacity of GST is 213.44 J/(kg·K).

## 3. Results and Discussion

### 3.1. Optical Simulation

**Figure 2:**
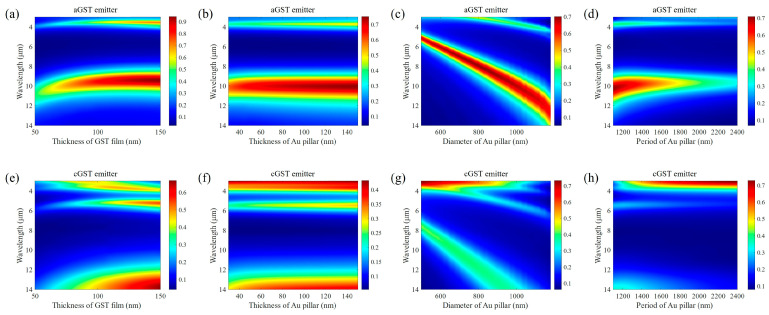
Dependence of the aGST emitter’s emission spectra on (**a**) the thickness of GST film t_1_, (**b**) the thickness of Au pillar t_2_, (**c**) the diameter of Au pillar d, and (**d**) the period of Au pillar p. Dependence of the cGST emitter’s emission spectra on (**e**) the thickness of GST film t_1_, (**f**) the thickness of Au pillar t_2_, (**g**) the diameter of Au pillar d, and (**h**) the period of Au pillar p. For each subgraph, only the parameter to be swept changes, while the other parameters remain at their initial values.

The initial structure parameters of the thermal emitter are set as t_1_ = 50 nm, t_2_ = 80 nm, t_3_ = 50 nm, t_4_ = 50 nm, d = 1 μm, and p = 1.2 μm. The emissivity spectra of the aGST emitter and cGST emitter obtained by sweeping t_2_, t_1_, d, and p are shown in Figure 2. For each sweeping, the parameters are fixed to their initial values except for the one to be studied. As the thickness of the GST film increases, the peak of the emissivity spectrum in 8–14 μm becomes higher. Simultaneously, the peak wavelength slightly shifts to a short wavelength. When the thickness of GST is over 80 nm, the emission peaks of the aGST emitter are larger than 0.7, whereas thick GST also brings large emissivity for the cGST emitter in 8–14 μm, causing a small variation range in the emissivity. The positions of the emission peaks are almost independent of the thickness of the Au pillar, but the emission peaks become a little larger with an increase in Au thickness. The emission peaks of both emitters undergo obvious redshift as the diameter of the Au pillar increases, and the peaks of the aGST emitter are higher than those of the cGST emitter in 8–14 μm. The emission peaks of both emitters become higher as the period of the Au pillar tends to its diameter. Hence, to acquire as large a difference as possible in emission spectra between the aGST emitter and cGST emitter in the 8–14 μm waveband, the thickness of GST film should be at least 80 nm, and the diameter of the Au pillar should be in a range of 0.8–1.2 μm. Additionally, the period of the Au pillar should be close to its diameter.

**Figure 3:**
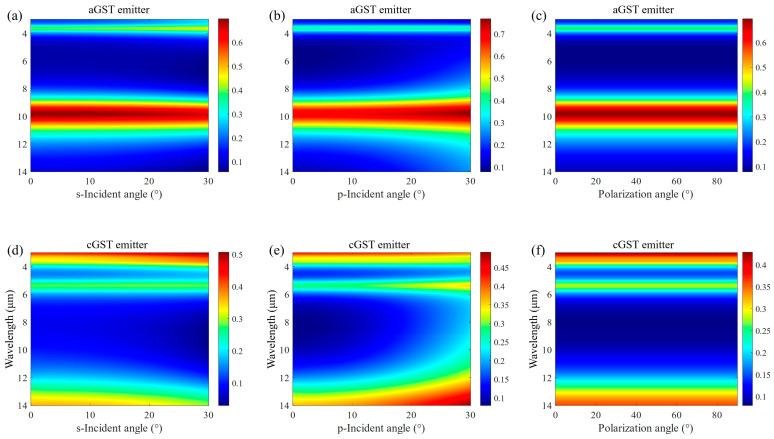
Influence of the incident angle and polarization angle on emission spectra. The aGST emitter’s emission spectra vary with (**a**) incident angles at s polarization (s-Incident angle), (**b**) incident angles at p polarization (p-Incident angle), and (**c**) polarization angles at normal incident. The cGST emitter’s emission spectra vary with (**d**) incident angles at s polarization (s-Incident angle), (**e**) incident angles at p polarization (p-Incident angle), and (**f**) polarization angles at normal incident.

The influence of the incident angle and polarization angle on the emission spectra of both emitters is also studied, as shown in Figure 3. It can be found that when the incident angle changes in a range of 0–30°, the spectral emissivity remains almost constant for the aGST emitter at both s and p polarization, whereas the incident angle insensitive range for the cGST emitter is only about 15°. When the incident angle is over 15°, the emissivity becomes weak for s polarization and strong for p polarization in 8–14 μm. Even so, the condition for dynamic infrared thermal camouflage can be met because the emissivity difference between the aGST emitter and cGST emitter is still very clear. Since our proposed MIM thermal emitter has a cylindrical symmetric structure, the polarization angle at normal incident theoretically does not affect the emission spectrum. It is verified in Figure 3c,f. Therefore, the thermal camouflage performance of the proposed MIM thermal emitter has a large incident angle tolerance and polarization insensitivity.

### 3.2. Thermal Simulation

The spectral emissivity of the GST emitter depends on the magnetic resonance performance of the MIM plasmonic metasurface, which is sensitive to the dielectric property of GST [31]. Hence, controlling the crystallization fraction of GST by electrical pulses to change its effective refractive index will provide a feasible method to tune the emission spectrum of the GST emitter. Generally speaking, the realization of GST amorphization is more difficult than its crystallization [30,40] because the amorphization not only requires a temperature over the melting temperature (900 K) but also needs a quench rate not less than 1.6 × 10⁹ K/s in the recrystallization temperature range (560–660 K) to reduce recrystallization probability [37]. In simulations and experiments, a long low-voltage pulse is usually applied to trigger crystallization via Joule heating, while a short high-voltage pulse is used to reamorphize GST via a melt–quench process. Additionally, the thermal conductivity of aGST is lower than that of cGST, thus rendering its heat dissipation capability weaker. Considering the aforementioned factors, only the melt–quench results of GST under a single electrical pulse are given in this work.

**Figure 4:**
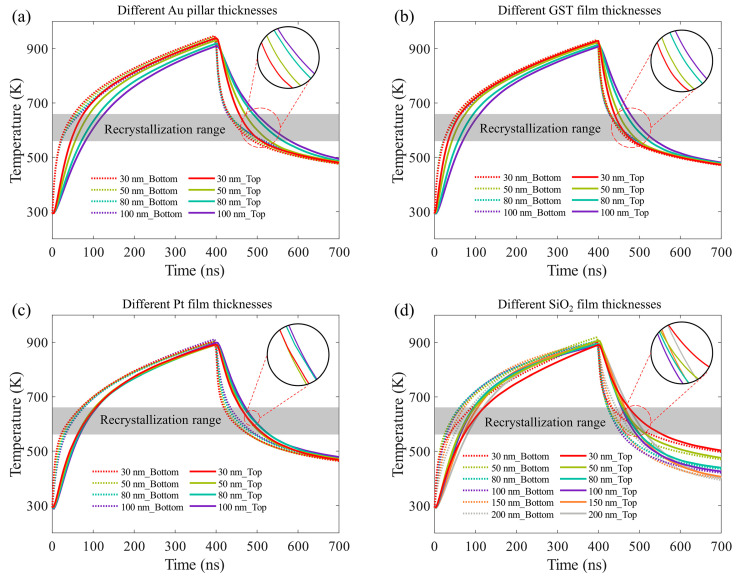
Simulated temperature evolution in GST under a 400 ns electrical pulse. Temperature variation curves at the bottom and top surfaces of GST with different (**a**) Au pillar thicknesses, (**b**) GST film thicknesses, (**c**) Pt film thicknesses, and (**d**) SiO_2_ film thicknesses.

In simulations, the temperature variation curves at the bottom and top surfaces of GST were recorded after the excitation of a 400 ns electrical pulse. To investigate the influence of structural parameters, we swept the thickness of the Au pillar, GST layer, Pt layer, and SiO_2_ layer, as shown in Figure 4. For the sake of comparison, the voltage of the electrical pulse was finely adjusted for each simulation so that the maximum temperature at 400 ns is just over 900 K. Moreover, the recrystallization temperature range (560–660 K) is drawn in Figure 4 for better comparison of the quench rate. Here, the quench rate is defined as the average falling rate of temperature in the recrystallization temperature range. It is clear that the rising and falling edges of the temperature curves at the bottom GST surface are faster than those at the top GST surface, due to the fact that the bottom GST surface is closer to the Pt heater and cold Si substrate. The difference becomes more and more obvious with an increase in the GST thickness, as shown in Figure 4b. This is also applied to the Au pillar thickness, depicted in Figure 4a. Because of its large thermal conductivity, Au absorbs some heat energy during the heating process and releases it during the quenching process, delaying the temperature change at the top GST surface. Therefore, the thicker the Au pillar and GST film are, the slower the change in temperature at the top GST surface is.

For the Pt film, the thickness has little impact on the temperature evolution in GST, due to its high thermal conductivity, as shown in Figure 4c. However, a thinner Pt film will require a higher voltage to reach the same temperature and have a higher risk of being burned out. In addition, since Pt also serves as a reflector in the MIM configuration, it is necessary to confirm whether all infrared emissions are reflected. Other simulations indicate that a 50 nm thick Pt is sufficient to reflect the majority of infrared emission. The SiO_2_ film with low thermal conductivity as a thermal insulation layer should take into account both heating efficiency and rapid quenching. A thick SiO_2_ film can improve the insulation effect and reduce energy consumption but hinders the energy dissipation to the substrate and leads to a slow quench rate. Conversely, a thin SiO_2_ film will require high voltage to raise the temperature over 900 K. At the same time, the substrate will be also heated to a relatively high temperature, resulting in a slow quench rate. From the simulation results in Figure 4d, it is clear that the quench rate is the fastest when the thickness of the SiO_2_ film is 100 nm. Combined with the optical simulation results, we choose the following structural parameters for the GST emitter: t_1_ = 50 nm, t_2_ = 80 nm, t_3_ = 50 nm, and t_4_ = 100 nm. The calculated quench rates at the bottom and top surface of GST are 3.13 × 10^9^ K/s and 3.07 × 10^9^ K/s, respectively, which satisfies the requirement of amorphization.

### 3.3. Thermal Camouflage Application

**Figure 5:**
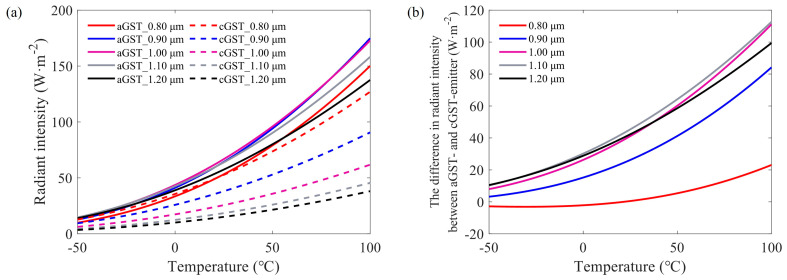
(**a**) The radiant intensity of aGST and cGST emitters with different Au pillar diameters. (**b**) The difference in radiant intensity between aGST and cGST emitters with the same diameter.

To further optimize the diameter of the Au pillar, we calculated the radiant intensity of aGST- and cGST-emitter with different Au pillar diameters using Equation (4) in a temperature range of −50 °C to 100 °C, shown in Figure 5a. For each calculation, the spectral emissivity was obtained at the condition that the period is 100 nm larger than the diameter. Figure 5b displays the difference in radiant intensity between aGST- and cGST-emitter with the same diameter. It can be seen that the difference is the largest for the Au pillar diameter of 1.1 μm. Then, the GST-emitter under this situation will have the widest range of background temperatures that thermal camouflage can be realized.

To investigate the emissivity characteristics of the MIM thermal emitter at various intermediate phases of GST, the effective permittivity *ε_eff_*(λ) of GST was calculated via the effective medium theory grounded on the Lorentz–Lorenz relation [41,42]:

(5) $$\frac{\varepsilon_{eff}(\lambda )-1}{\varepsilon_{eff}(\lambda )+2}=m\times \frac{\varepsilon_{c}(\lambda )-1}{\varepsilon_{c}(\lambda )+2}+(1-m)\times \frac{\varepsilon_{a}(\lambda )-1}{\varepsilon_{a}(\lambda )+2}$$

where *m* represents the crystallization fraction of GST, and *ε_a_*(λ) and *ε_c_*(λ) denote the permittivity of the amorphous and crystalline GST, respectively.

**Figure 6:**
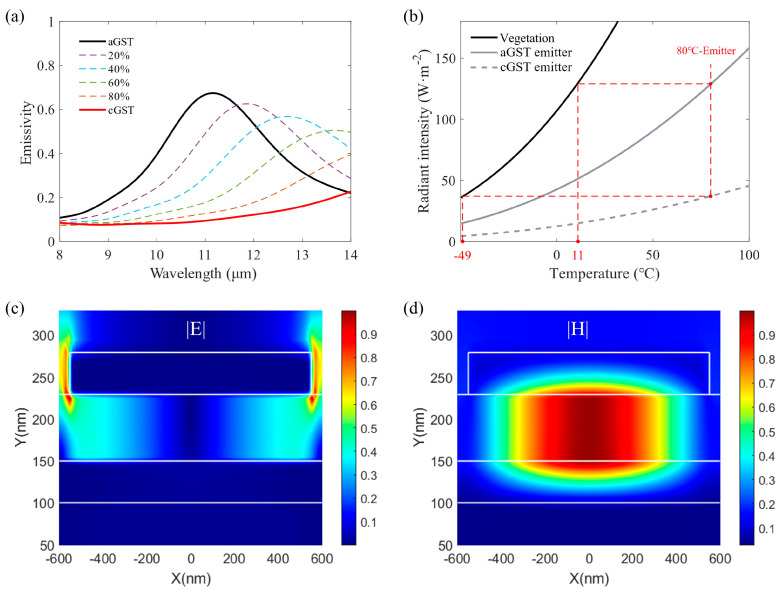
(**a**) Emissivity spectra of the GST emitter with different GST crystallization fractions. (**b**) Radiant intensity of the aGST or cGST emitter at different temperatures under a vegetation background. (**c**) Normalized amplitude distributions of the electric field |E| of the aGST emitter at 11 μm. (**d**) Normalized amplitude of the magnetic field |H| of the aGST emitter at 11 μm.

Based on obtained structural parameters, the spectral emissivity of the GST emitter with different crystallization fractions was simulated using the permittivity of the intermediate phase of GST, as shown in Figure 6a. The obvious redshift of emissivity peaks with the increase in the crystallization fraction portends that the radiant intensity has a large modulation range, and dynamic thermal camouflage can be achieved in a large background temperature range.

To verify this prediction, we chose a vegetation background as an example, whose emissivity (ε = 0.97) is comparable to that of the majority of natural backgrounds (ε = 0.85–0.98). Figure 6b plots the radiant intensity of the vegetation background (ε = 0.97) and GST emitter with different temperatures. The same radiant intensity means the same radiant temperature in thermal imaging cameras. Therefore, the target covered by GST emitters can blend into various vegetation backgrounds by tuning the crystallization fraction of GST. For example, an 80 °C emitter can achieve thermal camouflage in a vegetation background with a temperature range of −49 °C to 11 °C. Therefore, our proposed GST-based MIM thermal emitter has been theoretically demonstrated to be capable of blending into the environmental background with a large temperature variation range, allowing for adaptive dynamic infrared thermal camouflage.

To explore the underlying physical mechanisms of the emissivity peak at 11 μm, the normalized amplitude distributions of the electric field and magnetic field of the aGST emitter are displayed in Figure 6c,d. The electric field is mainly located on both sides of the Au pillar and has a node in the GST layer, which is consistent with the characteristics of an electric dipole. The magnetic field concentrates in the GST layer and has no node, which is a magnetic dipole or fundamental magnetic resonance [43]. Moreover, a partial field energy is dispersed in the air above the metasurface, leading to the emissivity less than one.

## 4. Conclusions

In summary, the structural optimization of an electrically controlled tunable MIM plasmonic thermal emitter based on GST was carried out. Combining the results of optical and thermal simulation, in order to achieve a large difference of spectral emissivity in 8–14 μm and a fast quench rate for the amorphization of GST, the chosen structural parameters are as follows: t_1_ = 50 nm, t_2_ = 80 nm, t_3_ = 50 nm, t_4_ = 100 nm, d = 1.1 μm, and p = 1.2 μm. The quench rates at the bottom and top surfaces of GST are up to 3.13 × 10^9^ K/s and 3.07 × 10^9^ K/s, respectively, fully sufficient to realize GST amorphization. It is demonstrated that this GST emitter can achieve dynamic thermal camouflage in a wide temperature range. For example, the emitter at 80 °C can blend into a vegetation background of −49 °C to 11 °C. Therefore, by optical and thermal models, the proposed GST-based MIM thermal emitter was proven to exhibit a large radiant intensity tuning range, wide incident angle tolerance, polarization insensitivity, considerable quench rate, and dynamic thermal camouflage capability. It is anticipated that this work will pave the way for the widespread application of GST emitters in infrared emission management and dynamic thermal camouflage.

## Data Availability

Data are contained within the article.
